# Laparoscopic Repair of Bochdalek Hernia: A Rare Presentation of Abdominal Pain in the Elderly

**DOI:** 10.1155/2023/5361609

**Published:** 2023-06-30

**Authors:** Christopher Steen, Jia Hui Lee, Enoch Wong, Sean Mackay

**Affiliations:** ^1^Monash University, Melbourne, VIC, Australia; ^2^Department of Surgery, Eastern Health, Box Hill, VIC, Australia

## Abstract

Bochdalek hernias (BHs) are rare, and the presentation, diagnosis, and management of them can be complex. We present a 70-year-old man presenting with left flank pain who underwent a successful laparoscopic repair of BH with mesh placement.

## 1. Introduction

One of the many achievements of anatomist Vincent Bochdalek was to describe a peculiar hernia that arose as an error of diaphragmatic development. It is known as the ‘Bochdalek hernia' (BH), this congenital diaphragmatic hernia is the protrusion of tissue through a defect in the lumbocostal triangle of the diaphragm, also known as the foramen of Bochdalek. This foramen is a result of the improper fusion of the septum transversum and the pleuroperitoneal folds during the development of the hernia in gestation [1].

## 2. Case Presentation

**Figure 1:**
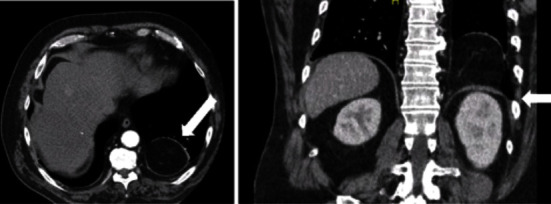
Axial and coronal slice CT scans depicting BH.

A 70-year-old man presented with increasing pain in the left flank on a background of a known BH. The hernia was discovered five years before and managed non-operatively as the patient lived rurally and had been asymptomatic. Contemporary imaging via computed tomography (CT) demonstrated that the hernia was now larger, and that there was a 15 mm defect in the posterior hemidiaphragm on the left side, with herniation of intra-abdominal fat, most likely from the retroperitoneum (Figure 1). The herniation measured at least 14 mm craniocaudally and about 66 mm in maximal transverse dimension.

**Figure 2:**
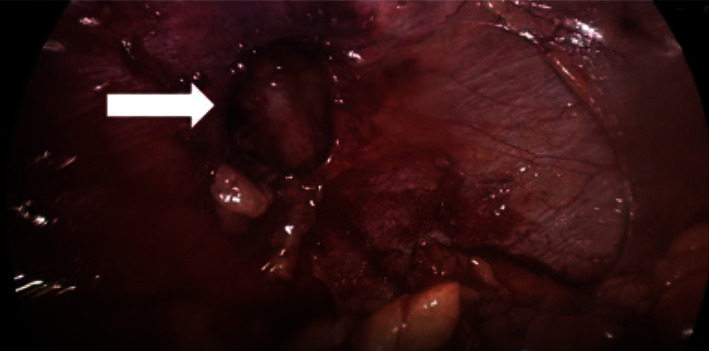
Intraoperative photo depicting defect in diaphragm following reduction of contents.

**Figure 3:**
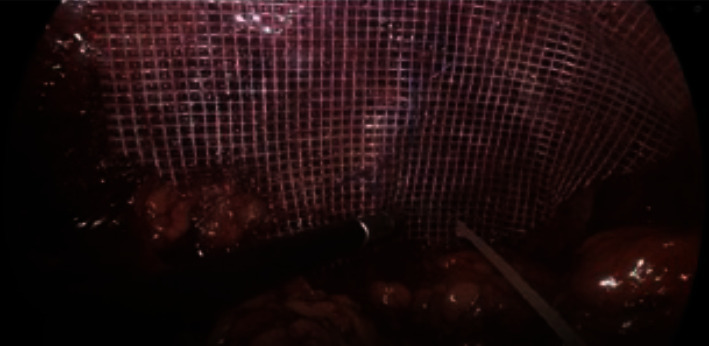
Intraoperative photo showing closure with prolene sutures and fixation of polyester mesh.

Given the patient was having ongoing back and left flank pain, likely due to strangulation of the herniated intra-abdominal fat, a laparoscopic diaphragmatic hernia repair of the foramen of Bochdalek was performed. Following the establishment of pneumoperitoneum and safe laparoscopic port placement, the short gastric vessels were divided to enter the lesser sac. The lateral and superior borders of the spleen were mobilised to improve the exposure of the diaphragm. The hernia was identified; the main findings were a small defect approximately 3 cm × 3 cm over the left dome of the diaphragm behind the left kidney, containing fat (Figure 2), which was reduced. The defect was closed with interrupted prolene sutures. A polyester mesh was placed and fixated with surgical glue (Figure 3). The patient went on to make a full recovery and was eventually discharged from outpatient surgical follow-up.

## 3. Discussion

The incidence of BH in infants is approximately 1 in 4000 [2]. In contrast, the incidence of BH in adults is estimated to be as low as 1 in 7000 [3]. One reason that may explain this difference is that a large foramen of Bochdalek may lead to herniation during prenatal development, which results in presentation shortly after birth due to compression-induced pulmonary hypoplasia [4]. A small foramen, a limiting pleural sac, or an omental plug in the defect may explain why some are present later in adulthood [5].

Asymptomatic BH occurs in approximately 0.17% of the adult population [5]. Otherwise, symptoms are variable. Common complaints are pain or discomfort in the chest and abdomen and exertional dyspnoea. Severe pain with nausea and vomiting suggests incarceration or strangulation. The main precipitants are physical exertion, pregnancy, defecation, or exacerbation of asthma. Abdominal symptoms generally occur first, followed by respiratory symptoms [5].

The diagnosis of BH is challenging due to its rarity and its non-specific presentation in adulthood [3].

Diagnosis may be obtained from plain radiographs or CT. A chest X-ray may demonstrate gas and organs above the diaphragm, whereas a CT is accurate in identifying diaphragmatic defects and signs of ischaemia [5]. The usual CT findings are fat or soft tissue over the upper posterolateral surface of the diaphragm, or a mass next to the defect [6]. If a BH is suspected then it is recommended that a CT with contrast should be performed due to its sensitivity (50–78%) and specificity (100%) in visualising focal diaphragmatic defects [3] and identifying strangulation [1].

Due to its presentation, delayed and missed diagnosis are common and can result in lethal complications. Once discovered, patients should undergo surgical repair as soon as possible, as mortality and complications are as high as 32% in emergency surgery, whereas the mortality rate for elective surgery is less than 3%. This makes early diagnosis vital in reducing the morbidity and mortality of the disease [7].

The main complications of BH are obstruction, strangulation, perforation, and other coexisting anomalies. It was noted that up to 18% of cases were complicated by life-threatening strangulation [1]. Given that strangulation rates of inguinal hernias are as low as 0.3%, there is a noteworthy risk of leaving BH untreated [8].

In the literature, both transthoracic and transabdominal approaches to surgical repair have led to similar outcomes [1]. Laparotomy was the most common approach and was performed in 38% of cases. Laparoscopic repair is increasing in popularity and can be performed with short hospital stays and complication rates as low as 7% [9].

## 4. Conclusion

Given the potential complications, this study aims to educate surgeons about BH, especially since it is an uncommon diagnosis that can present non-specifically. Surgeons should consider a lower threshold for operating on these hernias in the elective setting to prevent the occurrence of avoidable complications.

## Data Availability

All patient relevant data are available within the article.
